# Effect of Ta Additions on the Microstructure, Damping, and Shape Memory Behaviour of Prealloyed Cu-Al-Ni Shape Memory Alloys

**DOI:** 10.1155/2017/1789454

**Published:** 2017-01-11

**Authors:** Safaa N. Saud, E. Hamzah, H. R. Bakhsheshi-Rad, T. Abubakar

**Affiliations:** ^1^Faculty of Information Science and Engineering, Management and Science University, 40100 Shah Alam, Malaysia; ^2^Faculty of Mechanical Engineering, Universiti Teknologi Malaysia, 81310 UTM Johor Bahru, Johor, Malaysia; ^3^Advanced Materials Research Center, Department of Materials Engineering, Islamic Azad University, Najafabad Branch, Najafabad, Iran

## Abstract

The influence of Ta additions on the microstructure and properties of Cu-Al-Ni shape memory alloys was investigated in this paper. The addition of Ta significantly affects the green and porosity densities; the minimum percentage of porosity was observed with the modified prealloyed Cu-Al-Ni-2.0 wt.% Ta. The phase transformation temperatures were shifted towards the highest values after Ta was added. Based on the damping capacity results, the alloy of Cu-Al-Ni-3.0 wt.% Ta has very high internal friction with the maximum equivalent internal friction value twice as high as that of the prealloyed Cu-Al-Ni SMA. Moreover, the prealloyed Cu-Al-Ni SMAs with the addition of 2.0 wt.% Ta exhibited the highest shape recovery ratio in the first cycle (i.e., 100% recovery), and when the number of cycles is increased, this ratio tends to decrease. On the other hand, the modified alloys with 1.0 and 3.0 wt.% Ta implied a linear increment in the shape recovery ratio with increasing number of cycles. Polarization tests in NaCl solution showed that the corrosion resistance of Cu-Al-Ni-Ta SMA improved with escalating Ta concentration as shown by lower corrosion current densities, higher corrosion potential, and formation of stable passive film.

## 1. Introduction

Cu-based shape memory alloys are being considered as a prospective material for applications including high-damping capacity material, sensors, and actuators. Particularly, Cu-Al-Ni alloys are revealed to be appropriate for high-temperature applications due to their high thermal stability at increased temperatures, that is, above 373 K (100°C) [1, 2]. In contrast, the Cu-Al-Ni alloys produced by typical casting methods had to endure the issue of critical brittleness as a response for large grain sizes (around several millimeters) together with large elastic anisotropy [3–5]. For this reason, a number of efforts have aimed to improve the ductility of traditionally cast Cu-Al-Ni alloys via grain refining by the addition of a fourth element, which can include Ti, Zr, Mn, B, Y, V, Co, and rare earth metals [6–10]. The grain size of the resulting Cu-Al-Ni alloys was found to range from approximately 100 to 800 *μ*m. The grain refinement throughout alloying additions displayed enhancement in the mechanical properties of the Cu-Al-Ni alloys. On the other hand, the alloying elements are convenient to burn (atmospheric melting) or perhaps evaporate (vacuum melting) while performing the melting process, causing unrestrained transformation of temperature that can be significantly influenced by the composition of the alloy [11, 12]. As a result, the mechanical properties of such alloys remained unsatisfactory for most practical applications.

Several investigations have aimed to develop fine-grained Cu-Al-Ni alloys, with a grain size considerably less than 100 *μ*m, by using various powder metallurgy techniques [13, 14]. These kinds of techniques depended on the Cu-Al-Ni alloy powder, which was prepared by either an inert gas atomization process or mechanical alloying of elemental powders in a high-energy ball mill under inert gas atmosphere [15]. The mechanical alloying (MA) method [16, 17] is one of the most desired methods; it is indeed reported to be more affordable and also convenient for manufacturing applications. MA is predominantly beneficial to synthesize alloys with significant variance in melting temperatures. The method should prevent the mass loss of the component with the lower melting temperature due to the fact that the synthesis is performed near room temperature. MA is a powder processing approach involving a sequence of repeated welds with fracturing of the powder particles inside a mill. For the synthesis of prealloyed Cu-Al-Ni SMAs with free pores/cracks, numerous common sintering approaches have been studied [18–20]; however, most of these techniques involve lengthy sintering times (>3 h) and high sintering temperature (1050°C). Hence, there is strong interest from the PM industry to develop an innovative and superior sintering process with finer microstructures and improved physical and mechanical properties. This is where microwave technology promises to be advantageous [21, 22].

So far, the addition of tantalum has shown a significant effect on the microstructure, mechanical properties, and phase transformation temperature of shape memory alloys [23–25], due to its aptitude to reduce the transformation temperature, increase the thermal stability, and improve the strain recovery (*ε*_reco_) and residual strain (*ε*_res_) during thermal cycling. Therefore, it is suggested that Ta is a promising candidate for the alloying element to improve the shape memory property of SMAs [23]. On the other hand, the addition of Ta to Cu-Al-Ni SMAs has not been reported elsewhere; therefore, this research aims to investigate the influence of different amounts of Ta addition on phase transformation, mechanical properties, and corrosion behaviour of prealloyed powders of Cu-Al-Ni SMAs.

## 2. Experimental Procedure

### 2.1. Sample Preparation

In this research, the elemental powders of Cu, Al, and Ni, with Ta as an additional element, were prepared. The specification of the elemental powder and the initial powder mixture is shown in Table 1. These powders of Cu-Al-Ni-*x*Ta SMAs (*x* is 1.0, 2.0, and 3.0 wt.%) were prepared by mechanical alloying using planetary milling for 1 h at 300 rpm. For the mechanical alloying, a Retsch PM100 planetary ball mill with a zirconium oxide vial was used for 1 h to confirm the homogeneity of the powder. The rotation speed of the ball mill was 300 rpm, and ball to powder ratio was approximately 5 : 1 by weight.

The prealloyed powder was hot pressed into green samples with dimensions of *ϕ*15 mm × (L) 10 mm for the microstructural characterization and *ϕ*15 mm × (L) 30 mm for the mechanical test through a 10-ton hand-operated hydraulic press, and a single-act piston die of 15 mm diameter was utilized. The compaction process was carried out at a constant temperature of 300°C for 10 min; the temperature was maintained via an external heater tape connected to a thermoset to maintain the exact temperature. The green samples were placed into a 2.45 GHz, 0.3–3.0 kW consistently flexible microwave device (HAMiLab-V3, SYNOTHERM Corp.). The green samples were inserted inside an alumina sagger and covered with silicon carbide (SiC). The function of SiC is usually to serve as a microwave susceptor to enable the heating system as well as sintering of the green samples. The samples were sintered by microwave heating at a rate of 20°C/min to 900°C for 30 min. Argon gas with a purity of 99.995% was pumped into the microwave chamber throughout the sintering with the intent to protect against oxidation. To measure the temperature of all samples through the sintering process, a Raytek IR pyrometer was utilized. Prior to the microstructure characterization, the sintered samples were homogenized at 900°C for 30 min and directly quenched in water. Homogenization of the Cu-Al-Ni alloys at temperatures in the *β*-phase field followed by rapid cooling produces microstructures formed by metastable phases, which can result in martensitic transformation.

### 2.2. Porosity Calculation

The green porosity was calculated using the following equation [26, 27]:

(1) $$\begin{array}{c} P=\left\{\;1-\left(\;\frac{\rho_{g}}{\rho_{\text{th}}}\;\right)\;\right\}\times 100\%, \end{array}$$

where *ρ*_g_ is the green density and can be calculated by division of the calculated weight by the measured volume; and *ρ*_th_ is the theoretical density of the samples and can be calculated as follows [28]:

(2) ρth=ρ0Cu×at  %  Cu+ρ0Al×at  %  Al+ρ0Ni×at  %  Ni+ρ0Oxy×at  %  Oxy+ρ0additives×at  %  additives,

where *ρ*_0_^Cu^, *ρ*_0_^Al^, *ρ*_0_^Ni^, *ρ*_0_^Oxy^, and *ρ*_0_^additives^ are the theoretical densities of the base-alloy elements and additives.

### 2.3. Materials Characterization

The microstructure changes of the prealloyed and homogenized samples were investigated using a field emission-scanning electron microscope (FE-SEM), Zeiss-LEO Model 1530, operated at 10 kV coupled with energy-dispersive spectroscopy (EDS) operated at 10 kV. The results of EDS were indicated in accordance with a standardless semiquantitative analysis and an error bar in value of 5% was added to each reading. The phase and crystal structure were identified using a D5000 Siemens X-ray diffractometer fitted with a Cu K*α* X-ray source with a locked coupled mode, a 2*θ* range between 30° and 80°, and a 0.05°/s scanning step. The transformation temperatures of the mechanically alloyed Cu-Al-Ni alloy specimens with and without addition were evaluated via differential scanning calorimetry (DSC) at a heating/cooling rate of 10°C/min.

### 2.4. Mechanical Test

The internal fractions of the Cu-Al-Ni alloys with and without addition were evaluated by performing the damping test on the specimens in the martensitic state, wherein subsize test specimens with the dimension of 19 mm × 3 mm × 2 mm were prepared. The damping tests were carried out in a DMA Q800 dynamic mechanical analyzer in single-cantilever mode at a constant vibration frequency of 1 Hz and displacement of 0.05 mm, with a temperature range from 20°C to 300°C and a constant heating/cooling rate of 5°C/min. To measure the shape memory recovery of the prealloyed samples under multicycles, isothermal compressive loading and unloading were carried out at a tested temperature of 200°C, and, after each cycle, the sample was heated to *T* > *A*_*f*_, that is, ≈300°C, to obtain the shape recovery.

### 2.5. Corrosion Test

For potentiodynamic polarization (PDP) tests, cylindrical specimens with a surface area of 1 cm^2^ were prepared. PDP was carried out in an open-air glass cell containing 350 mL of 3 wt.% NaCl solution using a potentiostat (PARSTAT 2263 Princeton Applied Research). A three-electrode cell was used for the PDP tests, where a saturated calomel electrode (SCE) was used as the reference electrode, a graphite rod as the counter electrode, and an alloy specimen as the working electrode. The samples were immersed in the SBF for 1 h prior to the PDP test to establish the open-circuit potential. The samples were immersed in the NaCl solution for 1 h prior to the PDP test to establish the open-circuit potential. All experiments (*n* = 3, where n indicates the number of replicates) were carried out in the range between −250 mV in the cathodic direction and +500 mV in the anodic direction relative to the open-circuit potential at a constant scan rate of 0.167 mV/s. The polarization resistance (*R*_*P*_) was calculated according to the following equation [29, 30]:

(3) $$\begin{array}{c} R_{P}=\frac{\beta_{a}\beta_{c}}{2.3\left(\beta_{a}+\beta_{c}\right)i_{\text{corr}}}, \end{array}$$

where *i*_corr_ is corrosion current density, *β*_*c*_ is cathodic Tafel slope, and *β*_*a*_ is anodic Tafel slope of the specimens. The corrosion rate (*C*_*R*_) of the samples, obtained from the corrosion current density, was calculated according to [31]: ‎

(4) $$\begin{array}{c} C_{R}=22.85i_{\text{corr}}. \end{array}$$

Immersion testing was carried out according to ASTM G1-03. Specimens with a diameter of 10 mm and thickness of 10 mm were immersed in a beaker containing 200 mL of 3 wt.% NaCl solution for 30 days. The immersion tests were repeated at least once to verify the reproducibility of the results.

## 3. Results and Discussion

### 3.1. Green Density and Porosity

**Figure 1:**
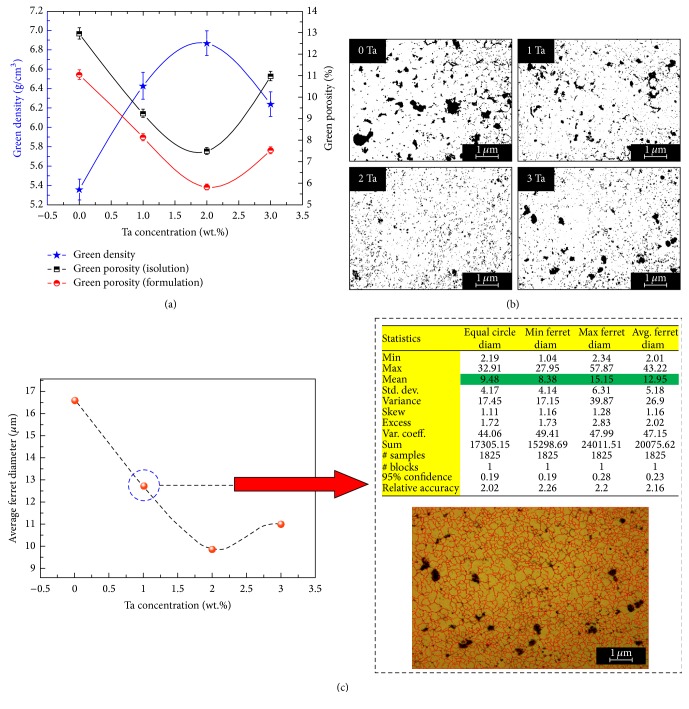
(a and b) Calculated green porosity and density of Cu-Al-Ni-*x*Ta SMA using formulation and image process contrast. (c) Grain size measurement in accordance with ASTM E112-12.

The variation of green density and porosity of the modified and unmodified alloys as a function of Ta amount is shown in Figures 1(a)–1(c). It can be clearly seen that the addition of Ta has produced a significant effect on the porosity density, in which the addition of 2.0 wt.% Ta led to an increase in the green density from 5.354 g/cm^3^ to 6.869 g/cm^3^, in consequence of reducing the green porosity from 12.96% to 7.5%. On the other hand, based on the micrographs in Figure 1(a), it was found that the Cu-Al-Ni SMA contains some semimicron-sized pores and that these pores were distributed randomly in the microstructure. The area fractions of the pores were calculated using image processing software known as *i*solution that also confirmed the same trend of decrement with the addition of Ta in which the lowest area fraction of pores was observed with 2.0 wt.% Ta addition. With further increase in Ta amount to 3.0 wt.%, the area fraction of pores increased, as shown in Figure 1(b). Utilizing *i*solution image processing software (*i*solution DT) and in accordance with the ASTM E112-12, the grain sizes of the modified and unmodified prealloyed samples were evaluated as indicated in Figure 1(c). It was observed that the grain size of the modified prealloyed samples significantly decreased, and the smallest grain size was indicated with the prealloyed sample of 2.0 wt.% Ta addition. This kind of reduction is mainly related to the effect of mechanical alloying, which also suggested that approximately 2 at.% Ta can be forced into the Cu lattice to form a supersaturated Cu-rich solid solution [32, 33] and produce a grain refinement. Darling et al. [32] have also revealed that the grain boundaries are more sensitive to the applied temperature of treatment and diffusion rate of Ta phase. In general, the grain size of Cu-Al-Ni SMA which is produced by conventional casting was determined to be 300–1400 *μ*m [34–36], even though the alloying elements and thermal treatments were applied.

### 3.2. Microstructural Investigations

**Figure 2:**
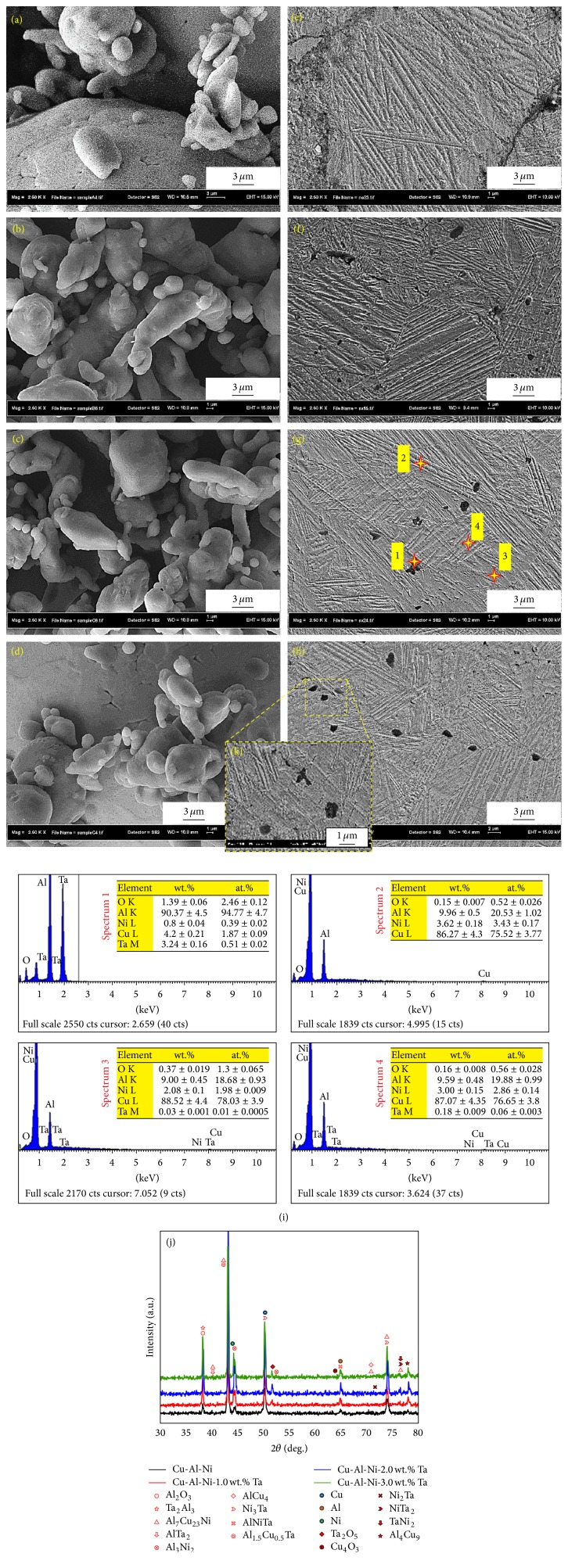
Morphologies of the milled powder and homogenized Cu-Al-Ni SMAs with and without Ta addition: ((a) and (e)) 0 wt.% Ta; ((b) and (f)) 1.0 wt.% Ta; ((c) and (g)) 2.0 wt.% Ta; ((d) and (h)) 3.0 wt.%; (i) EDS spectrums, (j) XRD diffraction of Cu-Al-Ni-2.0 wt.% Ta SMA; and (k) the magnified porosity with 3.0 wt.% Ta addition.

Figures 2(a)–2(h) show micrographs of prealloyed and homogenized Cu-Al-Ni SMAs associated with the chemical analysis of the homogenised samples. From the microstructure of prealloyed samples (see Figures 2(a)–2(d)), neck formation between the powder particles can be easily seen; these necks are caused by the cold working of the element powder that occurred during the mechanical alloying (ball-milling process). From the FE-SEM high-resolution images (Figures 2(e)–2(h)), it can be seen that there are two phases with different morphologies, plate-like and needle-like, with a self-accommodating configuration inside the merged grains. These phases are *β*_1_′ and *γ*_1_′ which are formed as thermally induced martensites and varied in terms of thickness and orientation after the addition of Ta. The *γ*_1_′ phase formed as a coarse variants/plate-like phase, while the *β*_1_′ phase formed as a needle-like phase between the *γ*_1_′ phases. The needle-like phase of *β*_1_′ martensite has a very pronounced thermoelastic behaviour, which can be attributed to its controlled growth in the self-accommodating groups [37]. However, when Ta was added, new phases were formed and the volume fraction of these precipitates varied according to the amount of Ta added. It is well known that Ta is an attractive element that causes the formation of second-phase/intermetallic compounds after addition [25, 38]. On the other hand, it was found that these precipitates were depleted in the Al/Ni matrix and, hence, the formation of *β*_1_′ martensite is promoted [36]. These precipitates accommodate the *γ*_1_′ and *β*_1_′ parent phases, and their accommodation is in a coherent or mostly semicoherent mode that depends on the precipitates' sizes and crystal-structure orientations relative to the parent phase [39]. Therefore, during the transformation of the precipitate into a single martensite variant after being surrounded by matrix, the precipitate leaves its place as a vacancy. However, the occurrence of an intrinsic deformation leads to a variety of other precipitates that are severely deformed during the transformation, and, thus, the precipitate maintains its own shape. It is well known that the microstructure and hence the mechanical behaviour of Cu-Al-Ni alloys change with the alloy composition and the processing routes to which the samples are subjected. The chemical compositions of the formed phases/precipitates in Cu-Al-Ni-2.0 wt.% Ta alloys were examined using EDS and are shown in Figure 2(i). It was found that the amount of elemental Ta in different microstructural locations was significantly changed based on the type of the formed phases/precipitates. Furthermore, the percentage of oxygen was found to decrease after the addition of Ta and homogenization.

The XRD patterns of the homogenized Cu-Al-Ni-*x*Ta SMAs with different percentages of Ta are presented in Figure 2(j). Indexing of these patterns shows that these only consist of martensite phases *γ*_1_′ and *β*_1_′ having a monoclinic structure as main phases along with some other precipitates/intermetallic compounds that are also formed after being homogenized at 900°C for 1 h. After the addition of Ta, the scanned peaks changed in terms of 2*θ* and intensity, which shows that the XRD patterns of Cu-Al-Ni SMA are sensitive to the amount of added Ta. On the other hand, the matrix of Cu-Al-Ni SMA as the predominant phase was always retained, even though Ta amounts varied.

### 3.3. Transformation Temperatures

**Figure 3:**
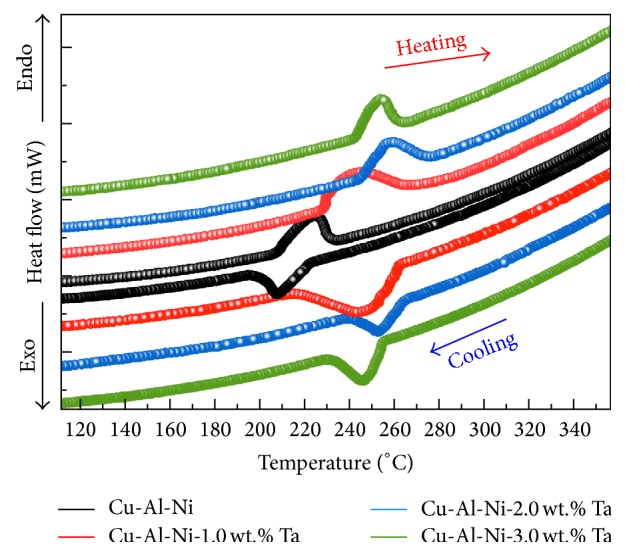
Endothermic and exothermic curves of Cu-Al-Ni-*x*Ta SMAs.

The endothermic and exothermic curves of the prealloyed samples of Cu-Al-Ni with and without Ta addition are shown in Figure 3 and the determined data are tabulated in Table 2. The endothermic curve during the heating represented the transformation of martensite to austenite phase, and this transformation is represented by the transformation temperatures of *A*_*s*_ and *A*_*f*_, austenite start and finish, respectively. Meanwhile, the exothermic curve during the cooling represented the transformation of austenite to martensite phase, which is represented by the transformation temperatures of *M*_*s*_ and *M*_*f*_, martensite start and finish, respectively. From Figure 3, it can be seen that the forward and backward transformations show one-step transformation in the modified and unmodified prealloyed samples due to the existence of a smooth single peak. The result reveals that the transformation temperatures are shifted towards higher temperatures. When the amount of Ta was approximately 1.0 wt.%, the transformation temperatures were slightly increased. Further increasing the amount of Ta to 2.0 wt.%, the transformation temperatures were rapidly increased compared with the unmodified alloy. However, the addition of 3.0 wt.% Ta led to decreasing the transformation temperatures compared with 2.0% Ta sample. It is well known that the transformation temperatures are mainly affected by the presence of precipitates, porosity, and their volume fraction [35, 40, 41]. Based on the micrographs in Figures 1(a) and 1(b), it is apparent that the percentage of porosity was increased as the percentage of Ta increased to 2.0 and 3.0 wt.%, and thus the transformation temperatures decreased.

### 3.4. Mechanical Properties

#### 3.4.1. Damping

**Figure 4:**
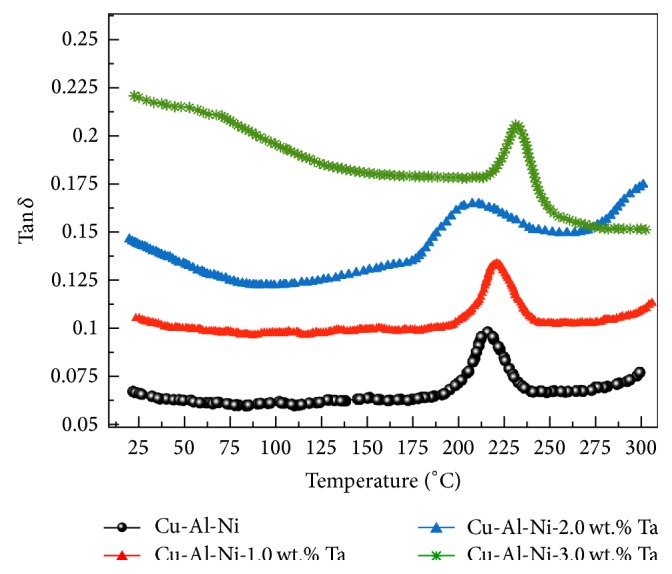
Internal friction heating curves of Cu-Al-Ni-*x*Ta SMAs.

In order to obtain the most accurate damping behaviour of the materials, Tan *δ* is the most suitable measurement, as it can give the ideal evolution of the signal over time. Within the low frequency, the peak of Tan *δ* during the martensitic transformation is mainly attributed to the transient internal friction [42, 43]. When the alloy is set at a certain temperature in an isothermal condition, the value of Tan *δ* is significantly decreased, which is associated with delaying the inherent internal friction and the intrinsic internal friction.  Figure 4 shows the internal friction (Tan *δ*) against the applied temperature, in which, based on Tan *δ* curve, there is only one peak observed for the modified and unmodified alloys which is related to the phase transition. It was found that the addition of Ta has significantly varied in the value of Tan *δ*, obtaining the highest value with the addition of 3.0 wt.% of Ta with respect to the base alloy. It can be also seen that the relaxation peak increased with increasing Ta amount. This is evidently explained by the presence of a multitude of Ta precipitates, which interfere with the movement of dislocations in the martensite phase and constitute the primary reason for the relaxation event.

In order to determine the influence of porosity on the damping behaviour and, based on previous studies [44, 45], an effective parameter, namely, equivalent internal friction (*Q*_eqv_^−1^), was proposed, and the value of this parameter can be determined using the following formula:

(5) $$\begin{array}{c} Q_{\text{eqv}}^{-1}=\frac{Q^{-1}}{\left(1-P\right)}, \end{array}$$

where *Q*^−1^ is the internal friction and *P* is the porosity. The results show that the equivalent internal friction was found to be in the consequence of 3.0 wt.% Ta > 2.0 wt.% Ta > 1.0 wt.% Ta > 0 wt.% Ta, in which the modified alloy with 3.0 wt.% Ta has highest *Q*_eqv_^−1^, which is almost twice that of the unmodified alloy. The mechanisms of internal friction for the Cu-Al-Ni SMAs are mainly related to the martensitic interfaces and transformation behaviour. During the mechanical loading and unloading and the thermal process, the martensite *↔* austenite phase transformation exhibits a dramatic lattice-softening phenomenon, and twin boundaries in the austenite phase are easier to form than in the martensite, which resulted in a higher damping capacity.

On the other hand, the presence of porosity may lead to making a difference in the elastic modulus and thermal expansion with respect to the matrix and thus cause a high thermal stress concentration around the pores [46]. In other words, that causes the pores to have a very complicated and inhomogeneous stress-strain state, in which this state causes a dilatation and distortion of pores and, hereafter, the dilatation and distortion energy increased. The dilatation and distortion of pores incorporate a number of processes including dislocation motion and formation which is certainly initialized in the material to relax stress concentration and then contributes to the dissipation of the elastic energy [47]. It is usually apparent that when the damping derives from a dislocation mechanism, it can be explained by strain amplitude reliance [48]. In addition, the martensitic transformation is also controlled slightly within the presence of porosity in SMAs and the index of energy dissipation, and, therefore, the value of Tan *δ* declines with increasing the density of porosity and decreasing of the grain size [47]. This statement can be supported by the lower number of IF values for the unmodified alloy that has the highest density of porosity, as indicated in Figures 1(a) and 1(b).

#### 3.4.2. Shape Memory Characteristics

**Figure 5:**
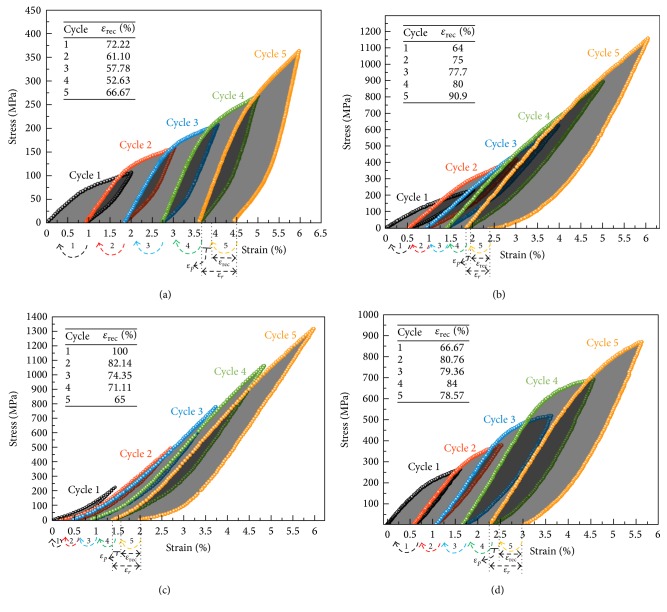
Stress-strain curves of Cu-Al-Ni-*x*Ta SMAs loaded and unloaded at 200°C and then preheated to 300°C. (a) Cu-Al-Ni and Cu-Al-Ni-*x*Ta SMAs with various Ta content: (b) 1.0, (c) 2.0, and (d) 3.0 wt.%.

Figures 5(a)–5(d) show the stress-strain curves measured for each training cycle of the modified and unmodified prealloyed Cu-Al-Ni SMAs. The stress is plotted against the prestrain that performed at a temperature of 200°C, and the indicated arrows within *x*-axis represent the strain recovery by shape memory effect at the corresponding cycle that was measured at a temperature of 300°C. In accordance with the s-s curves, it was found that the compressive stress increased after the addition of Ta, which has increased almost fourfold compared with the unmodified alloys. On the other hand, the shape memory recovery was also enhanced after the addition of Ta. The highest shape memory recovery was indicated with the modified alloy of 2.0 wt.% Ta (Figure 5(c)) which shows almost 100% recovery after being heated above *A*_*f*_ (i.e., 300°C) at the first cycle; however, as a further number of cycles were performed, this ratio tended to decrease. Both alloys of 1.0 wt.% and 3.0 wt.% show a linear increment in the strain recovery with increasing number of cycles, as observed in Figures 5(b) and 5(d). The fluctuations in the residual strain and recovery strain are mainly dependent on the direction and movement of martensite interfaces and dislocations [49, 50]. When the multitraining cycles were performed, the martensite variants were oriented unidirectionally, and, therefore, the plastic strain was stored in the samples. Within a certain number of cycles, the local stress is formed and results in increases in the density of dislocation and imperfections, which in opposite causes interlocking the dislocations and, thus, suppressing the movement of the martensite variants. As a result, the phase is unable to transform to the martensite phase and more residual plastic deformation is stored in the sample [51, 52]. On the other hand, increasing the number of thermal loading and unloading cycles can also result in increases in the volume fraction of the martensite variants. As a consequence, the structure of dislocations and martensitic variants remained constant during the training cycles, and, therefore, the shape memory effect decreased. As further numbers of training cycles are increased, associated with inducing a certain stress at a specific position, the density of dislocation reached the saturation level and led to the shape memory characteristics being stabilized.

### 3.5. Electrochemical and Immersion Test

**Figure 6:**
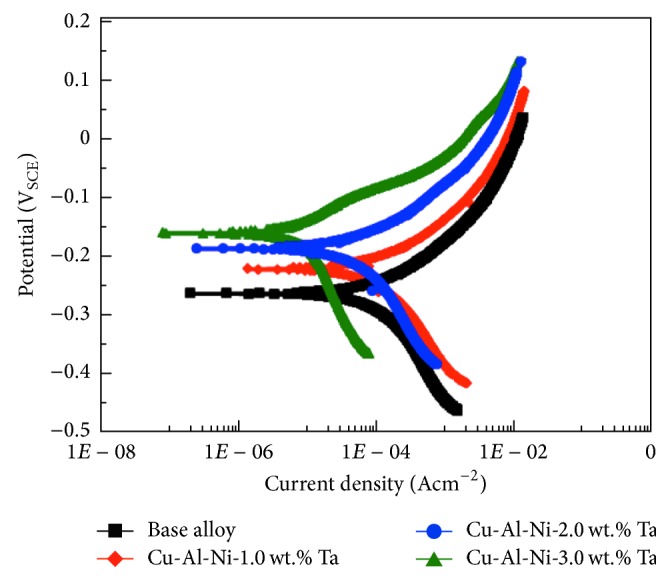
Potentiodynamic polarization curves of specimens in 3 wt.% NaCl solution. (a) black curve, Cu-Al-Ni and Cu-Al-Ni-Ta SMAs with various Ta content: (b) red curve, 1.0, (c) blue curve, 2.0, and (d) green curve, 3.0 wt.%.

Typical potentiodynamic polarization curves of base Cu-Al-Ni SMA and Cu-Al-Ni-*x*Ta (*x* = 1.0, 2.0, and 3.0 wt.%) SMAs are plotted in Figure 6. Corrosion potential (*E*_corr_), corrosion current density (*i*_corr_), and polarization resistance (*R*_*P*_) of the SMA samples are presented in Table 3. *E*_corr_ of SMA containing Ta was nobler compared with the base SMA sample. *E*_corr_ of the Cu-Al-Ni-1.0 wt.% Ta sample was approximately −220.5 mV_SCE_, whereas that of the base SMA sample was around −261.6 mV_SCE_. The Cu-Al-Ni-3.0 wt.% Ta sample evidently had more positive *E*_corr_ (−159.3 mV_SCE_) compared with the Cu-Al-Ni-2.0 wt.% Ta sample (−185.1 mV_SCE_). This illustrated that the addition of Ta to the base SMA sample ennobles the open-circuit potential. In fact, the SMA containing 3.0 wt.% Ta indicates the nobler pitting potential that causes a decline of pitting susceptibility and increases pitting corrosion resistance [53]. Polarization curves also display that *i*_corr_ of the base SMA and Cu-Al-Ni-1.0 wt.% Ta SMA was 117.6 and 78.4 *μ*A cm^−2^, respectively. The base SMA is not passivated and dissolves actively, forming a corrosion product film [54]. Thus, the Cu-Al-Ni-1.0 wt.% Ta SMA presented better corrosion resistance than that of the base SMA. Addition of 2.0 and 3.0 wt.% Ta to the base SMA reduced *i*_corr_ to 32.7 and 12.8 *μ*A cm^−2^, respectively. The higher corrosion resistance of the SMA containing higher Ta concentration is due to the rapid formation of a passive film with a highly protective quality and high uniformity [54].

**Figure 7:**
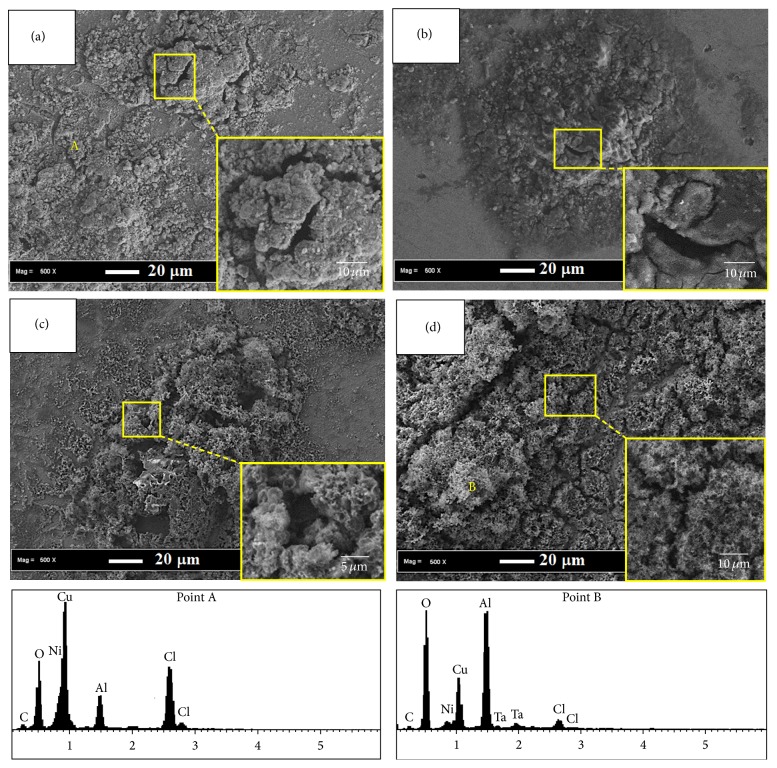
SEM micrographs of (a) Cu-Al-Ni and Cu-Al-Ni-*x*Ta SMAs with various Ta content: (b) 1.0, (c) 2.0, and (d) 3.0 wt.%; and corresponding EDS analyses of points A and B after immersion into 3 wt.% NaCl for 30 days.

From the curve, it can be observed that *β*_*a*_ is ~0.07 V/decade for the SMA containing Ta. This value is close to the theoretical and experimental value of 0.06 V/decade for copper in chloride media at room temperature [55]. *R*_*p*_ values indicated a positive influence of Ta additions on the corrosion behaviour of Cu-Al-Ni SMA alloy. Thus, *R*_*p*_ values increased from 4.57 to 7.18 KΩ cm^2^ when a small quantity of Ta (1.0 wt.%) was added to the base SMA. Further addition of 2.0–3.0 wt.% Ta to the base SMA leads to a significant increase of *R*_*p*_ value in the range of 18.64–34.14 KΩ cm^2^. This indicated that the addition of 3.0 wt.% Ta to base SMA led to the formation of a highly protective passive film even in aggressive chloride-containing solution [54]. In this regard, tantalum-containing alloys showed high corrosion resistance due to spontaneous passivation in aggressive media [56]. Tantalum addition to base SMA leads to a decreasing trend in corrosion rate (*C*_*R*_), particularly when 3.0 wt.% Ta was added. *C*_*R*_ values shown in Table 3 clearly indicate the corrosion rate in the following order: Cu-Al-Ni, Cu-Al-Ni-1.0 Ta, Cu-Al-Ni-2.0 Ta, and Cu-Al-Ni-3.0 Ta. The lowest corrosion rate of Cu-Al-Ni-3.0 Ta SMA is attributed to the presence of a high concentration of Ta ions in the corrosion product film, which has low solubility in aggressive chloride-containing solution [54]. In view of this, it was reported that tantalum is an essential element to improve the corrosion resistance of SMA in aggressive media [56]. Surface morphologies of the Cu-Al-Ni and Cu-Al-Ni-*x*Ta SMA after 30 days of immersion in NaCl solution are shown in Figure 7. Base Cu-Al-Ni SMA indicated small pits and a significant amount of surface cracking on the surface of the base SMA, which is due to dehydration after removal from the NaCl solution. The base alloy dissolves actively owing to the formation of the CuCl_2_^−^ complex anion [53]. From Figure 7(b), the presence of pitting and corrosion products can be observed on the surface of Cu-Al-Ni-1.0 Ta SMA. However, lower amounts of corrosion products were detected after the addition of 2.0 wt.% Ta to the base SMA (Figure 7(c)). SMA containing 3.0 wt.% Ta yielded the formation of dense uniform protective films enriched in tantalum ions that cover the entire SMA surface (Figure 7(d)) [57]. A plateau in the anodic polarization curve can clearly be seen, revealing the formation of passive film on the 3.0 wt.% Ta SMA surface [57]. However, Cu-Al-Ni and Cu-Al-Ni-1.0 Ta SMAs were not passivated and dissolved actively, indicating that the 2.0–3.0 wt.% Ta addition to the base SMA stabilizes the protective film. This suggests the presence of tantalum in a corrosion product such as tantalum oxyhydroxide film functioning as the effective barrier film. El-Moneim [57] showed that the presence of tantalum in single solid solution phase alloys suppresses the active dissolution process and enhances the protective quality of the passive film formed. EDS analysis (Point 1) shows high amounts of Cu, Cl, and O accompanied by low content of Ni, indicating the formation of copper compounds in the form of oxide or chloride and aluminum oxide. The presence of Ta (Point 2) further confirmed that tantalum is concentrated in the passive film. In this regard, Badawy et al. [58] reported that, in the corrosion product in ternary aluminum-containing copper alloys composed of two layers, the under layer is Cu_2_O. Al_2_O_3_·*x*H_2_O, the overlayer, is a mixture of Al_2_O_3_ and Cu_2_O. However, Montecinos and Simison [59] suggested that the corrosion product of the Cu-Al-Be SMA in chloride media is composed of Al_2_O_3_·H_2_O, Cu_2_O, CuO, (CuCO_3_·Cu(OH)_2_), and CuCl_2_. In view of this, the formation of protective tantalum-enriched film in addition to Al-dihydroxychloride, Cu-oxides, and Cu-chlorides is responsible for the high corrosion resistance of Cu-Al-Ni-Ta SMA.

## 4. Conclusions

The Cu-Al-Ni alloy as a potential type of shape memory alloy was successfully produced by powder metallurgy, mechanical alloying, and microwave sintering. The addition effects of different amounts of Ta on the microstructure, transformation temperature, damping capacity, shape memory effect, and corrosion behaviour were systematically investigated and the main conclusions are as follows:

1. After the microwave sintering at 900°C, the Ta particles were uniformly distributed in the matrix of Cu-Al-Ni, and different types of precipitates were formed in the binding domain between the Ta and Al/Ni phase.
2. The porosity density and grain size were reduced after the addition of Ta, in which the smallest grain size and lowest porosity were observed in prealloyed Cu-Al-Ni after being modified with 2.0 wt.% Ta.
3. The highest transformation temperatures and strain recovery by shape memory effect were indicated in prealloyed Cu-Al-Ni-2.0 wt.% Ta, while the highest internal friction was present in the prealloyed Cu-Al-Ni-3.0 wt.% Ta. These variations are mainly attributed to the density of porosity, grain refinement, and presence of precipitates, whereas these parameters significantly control the movement of martensite interfaces and dislocations, thus controlling the mechanical properties.
4. The electrochemical corrosion performance of the Cu-Al-Ni-Ta SMA was enhanced via increasing the Ta concentration. The result also indicated that a more stable passive oxide film containing tantalum oxyhydroxide formed on the surface of Cu-Al-Ni-3.0 Ta SMA, which resulted in better corrosion resistance compared with the other SMAs.

## Figures and Tables

**Table 1:** Specification of elemental powders and mixture.

Properties	Cu	Al	Ni	Ta
Size (*μ*m)	150 ± 7.5	25 ± 1.25	45 ± 1.2	45 ± 1.2
Purity (%)	99 ± 4.5	99 ± 4.5	99.5 ± 5	99.9 ± 5
Composition (wt.%)	83.5 − *x*	12.5 ± 0.7	4 ± 0.2	*x* (1.0, 2.0, and 3.0)

**Table 2:** Transformation temperature of Cu-Al-Ni SMA with and without Ta additions.

Alloy	Transformation temperatures (°C)			
	*A*_*s*_	*A*_*f*_	*M*_*s*_	*M*_*f*_
Cu-Al-Ni	207	235	226	196.25
Cu-Al-Ni-1.0 wt.% Ta	225.8	267.57	263	211.8
Cu-Al-Ni-2.0 wt.% Ta	242.7	277.4	265.6	237.9
Cu-Al-Ni-3.0 wt.% Ta	240.5	265.6	256.8	229

**Table 3:** Electrochemical parameters of ternary Cu-Al-Ni and quaternary Cu-Al-Ni-Ta SMAs in 3 wt.% NaCl solution obtained from the polarization test.

Alloy	Corrosion potential, *E*_corr_ (mV versus SCE)	Current density, *i*_corr_ (*μ*A/cm^2^)	Cathodic slope, *β*_*C*_ (mV/decade) versus SCE	Anodic slope, *β*_*a*_ (mV/decade) versus SCE	Polarization resistance, *R*_*P*_ (kΩcm^2^)	Corrosion rate, *C*_*R*_ (mm/year)
Cu−Al−Ni	−261.6	117.6	−211	78	4.57	2.68
Cu−Al−Ni−1.0 Ta	−220.5	78.4	−184	76	7.18	1.79
Cu−Al−Ni−2.0 Ta	−185.1	32.7	−148	72	18.64	0.74
Cu−Al−Ni−3.0 Ta	−159.3	12.8	−220	69	34.14	0.29
